# Spatial ecology of coyotes in the urbanizing landscape of the Cuyahoga Valley, Ohio

**DOI:** 10.1371/journal.pone.0227028

**Published:** 2019-12-30

**Authors:** Gregory A. Franckowiak, Marlo Perdicas, Gregory A. Smith

**Affiliations:** 1 Depatment of Biology, The University of Akron, Akron, Ohio, United States of America; 2 Summit Metro Parks, Akron, Ohio, United States of America; 3 Department of Biological Sciences, Kent State University at Stark, N. Canton, Ohio, United States of America; Urbino University, ITALY

## Abstract

Urban landscapes can present ecological challenges for wildlife species, yet many species survive, and even thrive, near dense human populations. Coyotes (*Canis latrans*), for example, have expanded their geographic range across North America and, as a result of their adaptability and behavioral flexibility, are now a common occupant of many urban areas in the United States. We investigated the spatial ecology of 27 coyotes fitted with Global Positioning System (GPS) telemetry collars radio-collared in the Cuyahoga Valley, Ohio. Our objectives were to quantify coyote space use, evaluate resource selection, and investigate coyote movement and activity patterns. To measure space use, we estimated home range (95%) and core area (50%) size of coyotes using the adaptive local convex hull (*a-*LoCoH) method. We found the mean (± SE) home range size of resident coyotes (4.7 ± 1.8 km^2^) was significantly smaller than ranges of transient coyotes (67.7 ± 89.6 km^2^). Similarly, mean (± SE) core area size of resident coyotes (0.9 ± 0.6 km^2^) was significantly smaller than core areas of transient coyotes (11.9 ± 16.7 km^2^). Home range and core area size of both resident and transient coyotes did not vary by sex, age, or season. For all coyotes, use of natural land cover was significantly greater than use of altered and developed land. When coyotes were using altered and developed land, GPS fixes were most common at night. Coyote movement patterns differed with respect to status, time period, and season; peaking during nighttime hours. A better understanding of coyote space use and movement within anthropogenic landscapes aids management of people, parks, and wildlife by providing the data necessary for research-based management decisions.

## Introduction

Increasing urbanization continues to impact natural ecosystems, including remnant habitats within urban areas as well as habitats on the periphery of city and town boundaries [1]. The expansion of urban lands now accelerates faster than lands set aside for parks and conservation; therefore, wildlife must adjust to human-dominated systems, or be excluded from those environments [2,3]. Consequently, many species are displaced by urbanization, while some survive, and even thrive near dense human populations [4].

Coyotes (*Canis latran*s) in North America are opportunistic carnivores that have adapted to the infringement of urbanization, while also dwelling in urban landscapes that were once unoccupied [5]. Many of these human-developed landscapes are comprised of city parks, wooded preserves, and water sources that coyotes can utilize as habitat [5–8]. Within these urbanized environments, examination of coyote diets have confirmed that coyotes consume both natural (e.g., deer, small mammals, and fruits) and anthropogenic foods (e.g., refuse, domestic pets, pet food, and cultivated plants) [9–14]. Moreover, coyotes have become a species of concern in human dominated areas, where they occasionally are involved in conflicts with people and pets [12,15], and carry transmittable diseases [7,12,16]. However, granted much negative attention is placed on coyotes, their presence regulates the numbers of other mesopredators, allowing for an increase in bird diversity [17], and can maintain or increase rodent species richness and diversity[18].

Urban coyote ecology has been studied in metropolitan areas across North America. The occurrence of coyotes in these human-dominated landscapes suggests that, while relying on the presence of natural habitats for much of their life history, they are adaptable in their use of the landscape [7]. Prior examination of coyote spatial and temporal use of urban landscapes has suggested selection for natural land covers within their home ranges, with individuals typically avoiding heavily developed areas [7,19–22]. In addition, coyotes in urbanized areas often shift to a more crepuscular and nocturnal activity pattern [20,21,23]. As observed with other wildlife species, this modified temporal behavior is likely a response to human presence [24–26]. In areas with large populations of both coyotes and humans, management strategies that are based on an understanding of coyote space use are most likely to be effective in reducing human-coyote conflicts.

Our objectives were to (1) quantify coyote space use (home range / core area size and land cover composition), (2) evaluate resource selection, and (3) investigate movement and activity patterns of coyotes trapped within the urbanizing landscape of the Cuyahoga Valley, Ohio. We predicted that resident coyote space use would be comparable to those of coyotes in previous studies of urban environments, and that space use would increase with increased development within those coyote ranges. Irrespective of home range and core area land cover composition, we expected the highest percentage of coyote telemetry locations to be found in natural lands as opposed to altered and developed land cover types. Following prior investigations that have shown coyotes to be more active during crepuscular and nighttime periods, we predicted that coyotes in our study would also be more active during these periods, spending more time in natural areas during diurnal periods, and more time in altered and developed lands during crepuscular and night time periods. This investigation will increase our understanding of how coyotes utilize a natural landscape that is heavily used by humans and is adjacent to large cities, allowing for comparisons of urban coyote ecology between urbanized areas. In particular, our study will increase our understanding of how coyotes utilize the landscape in an area where increasing urbanization is infringing on natural lands, providing research-based data for management decisions in this region.

## Materials and methods

### Ethics statement

Animal capture and handling protocols were approved by the approved by the University of Akron Institutional Animal Care and Use Committee (Approval #11-2B) and followed guidelines approved by the American Society of Mammalogists [27]. Coyotes are not an endangered species in Ohio. Any permissions needed were granted by the Summit Metro Parks, Cleveland Metroparks, the National Parks Service, and Ohio Department of Natural Resources’ Division of Wildlife.

### Study area

**Fig 1:**
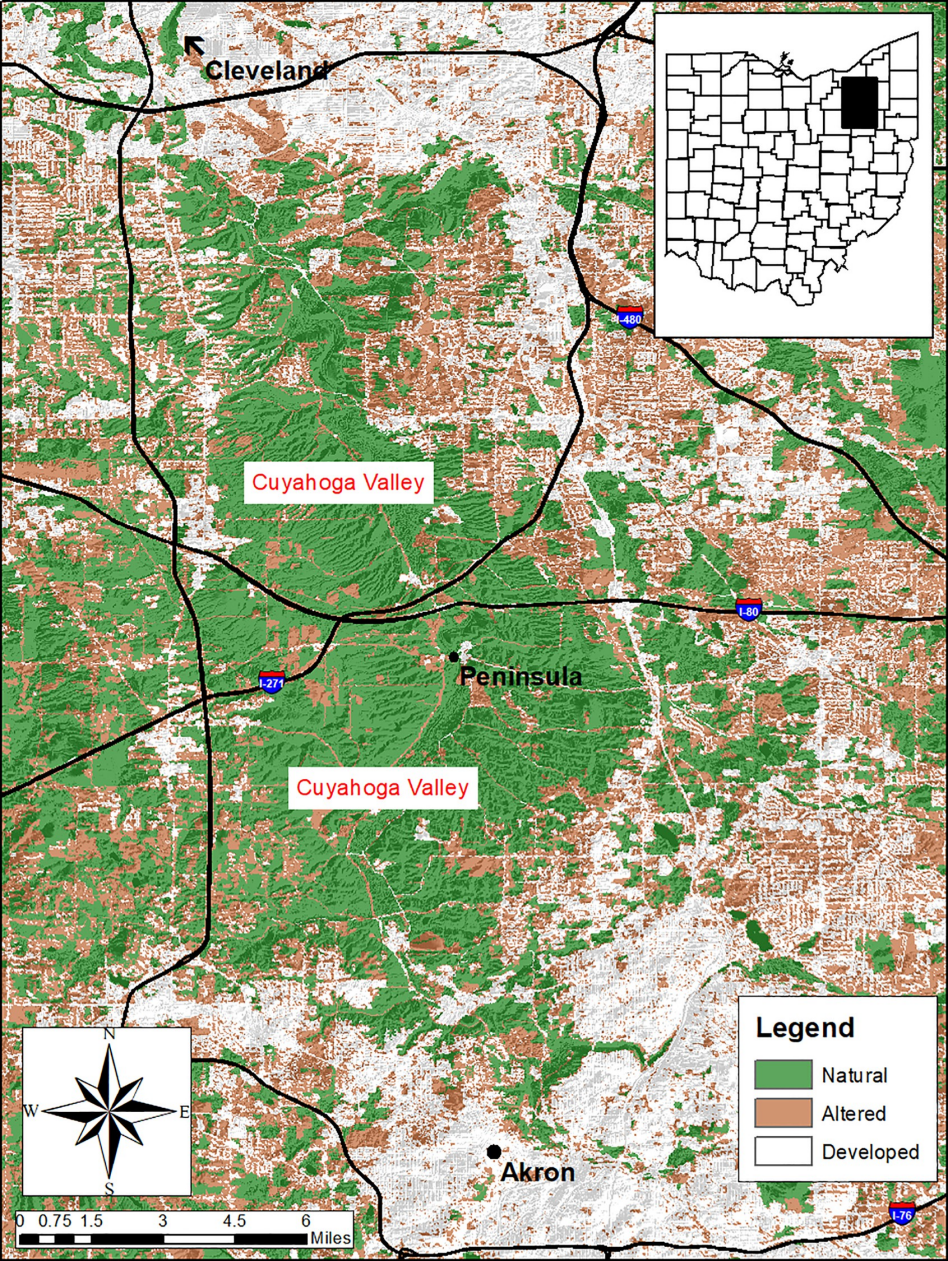
Map of the Cuyahoga Valley and 3 land cover categories, Ohio 2010–2012.

Our study was conducted in the Cuyahoga Valley, Ohio which included parts of Cuyahoga and Summit counties between the cities of Cleveland and Akron (41°19’23” N, 81°35’17” W; Fig 1). The study area is approximately 14,052 ha (34,723 acres) and the estimated human population is 1,280,122 and 541,781 for Cuyahoga (Cleveland) and Summit (Akron) counties, respectively [28]. The Cuyahoga Valley is bisected by the Cuyahoga River that extends from Akron, Ohio to Lake Erie. Much of the property in the valley is owned and operated by Cuyahoga Valley National Park and adjoining properties of Cleveland Metroparks and Summit Metro Parks. The Cuyahoga Valley is comprised of many roads, highways and recreational trails, and between the years 2010–2012 attracted an average of 2.3 million visitations per year [29]. Urban and suburban development and communities are found within the valley area and adjacent natural lands. Climate throughout the study was characterized by hot summers with temperatures exceeding 38°C and cold winters below -18°C, with monthly precipitation levels averaging 9.86 cm. Snow-fall occurred in the months of December through March and averaged 33.5 cm per month [30].

### Capture and telemetry

Coyotes were trapped within properties controlled by the National Park Service, Cleveland Metroparks, and Summit Metroparks during the months of October through February in 2010 through 2012. Public trails were closed for trapping, or traps were set far enough away from recreation sites to minimize risk to park guests and pets. Traps were checked daily. Coyotes were trapped with padded-leg-hold traps or cable restraints with stops to prevent injury. Coyotes were immobilized with Medetomidine (50 ug/kg) and Butorphanol (0.3 mg/kg). During processing, coyotes were weighed, sexed, aged by tooth wear [31], given a fecal exam, and a blood sample was taken to assess health conditions. Individuals received a numbered ear tag, pit tag, and were fitted with a Lotek WildCell global positioning satellite (GPS) collar (Lotek Wirelesss, Inc., Newmarket, Canada). Coyotes were released at the site of capture when fully recovered.

### Analysis

GPS fixes were collected from October 2010 thru November 2012. For the first year of data collection, fixes were collected every 1.5 h from 2000 h– 0800 h, and every five hours from 0800 h-2000 h. At random three-day intervals once per week, GPS locations were collected every 1.5 h from 0500 h-2000 h, and every five hours from 2000 h—0500 h. During the second year of data collection, diurnal fixes were collected every 3 h in order to increase the number of diurnal data points for analyses and more accurately monitor diurnal coyote locations.

Spatial data were analyzed using the ArcGIS 10.3 Geographical Information System (GIS)[32]. All data were analyzed in the UTM 17N Projected Coordinate System. For collected GPS data, 2D and non-validated point locations (those locations taken by less than five satellites) were removed from the analysis due to higher amounts of associated error [33].

At the time of immobilization coyotes were categorized as “juvenile” if they were < 1 year old, “subadult” if 1–2 years old, and “adult” if > 2 years of age [34,35]. However, in order to increase sample sizes for some analyses, coyotes were pooled by age class, where “adults” were considered >2 years of age and “young” were juveniles and subadults combined. We defined resident coyotes as individuals utilizing one defined area for ≥ 1 biological season, and transient coyotes as individuals exhibiting no permanent preference for a single area. Home ranges of transient coyotes often overlapped home ranges of residents. [36]. For seasonal analyses, seasons were classified into 3 periods: breeding (1 Jan-30 April), pup-rearing (1 May- 31 August), and dispersal (1 September- 31 December)[7]. Four time periods of the day were classified as: dawn (1 h before to 2 h after sunrise), day (2 h after sunrise to 1 h before sunset), dusk (1 h before to 2 h after sunset), and night (2 h after sunset to 1 h before sunrise) [37]. We determined sunset and sunrise times for each movement using information from http://aa.usno.navy.mil/data/docs/RS_OneYear.php.

### Space use estimates

Space use estimates were calculated using the adaptive local convex hull method (*a*-LoCoH) [38], using the isopleth nearest to 95% to estimate the home range and the isopleth nearest to 50% to calculate core area [39]. All isopleths were calculated using the AdehabitatHR package version 0.4.15 [40] in RStudio version 1.1.442 [41]. The value of *a* was established by visually inspecting the maps using the “minimum spurious hole covering” technique, which ensures the physical features that cannot form part of the home range (e.g. lakes) are omitted from the *a*-LoCoH estimate [42]. Composite (comprised of all data for each individual) and seasonal space use estimates were calculated in km^2^. In addition, we calculated 95% minimum convex polygons (MCP) [43] and included descriptive statistics. This method is commonly used and thus allows comparison of results across studies [7,16,22,34]. MCPs were also calculated in km^2^ using the AdehabitatHR package.

Home range and core area estimates were calculated by status, sex, age, and season. Composite space use was estimated for coyotes that were monitored for over half of a biological season. All analyses were performed in RStudio, with P < 0.05 considered statistically significant. We used *t*-tests to determine differences in home range and core area sizes by status, between sexes within status groups, and age. We used analysis of variance (ANOVA) to determine differences in seasonal space use. Differences in coyote age were only tested for home range and core area size in order to allow for comparison across studies and were not included as a factor for other analyses. All home range and core area sizes were tested for normality using a ‘quantile-quantile’ plot in RStudio. Space use estimates were log-transformed in order to meet distributional assumptions for all analyses.

Land cover composition refers to the percentage of each land cover type available to or used by an animal. We estimated the percentage of land cover types within each coyote’s composite and seasonal home range in ArcGIS 10.3 using land use coverage from the 2011 National Land Cover Dataset [44]. We reclassified coverage from the original 15 land cover types and summed the proportions of varying coverage types within home range and core areas, consolidating land cover types into three broad categories: developed, altered, and natural land covers[20] (Table 1). The percentage of each land cover type was measured using the ‘Tabulate Area Tool’ in ArcGIS. The numbers of cells of each land cover type were counted within the home range each coyote, and a percentage of each land cover type was calculated as a function of the total number of all cells. In addition, we determined coyote use of available land cover types by intersecting coyote GPS fixes with land covers in ArcGIS. To determine distance of coyote locations to the nearest road, GPS fixes were spatially joined with road data available from the Ohio Department of Transportation [45] using ArcGIS 10.3.

**Table 1:** Land use, descriptions, and classifications of land covers available to coyotes (*Canis latrans*) radio-collared in the Cuyahoga Valley, Ohio, 2010–2012.

Land Use Type	Description	Classification
Low Development	Combination of constructed materials and vegetation. Impervious surfaces account for 20% to 49% percent of total cover. These areas most commonly include single-family housing units.	Developed
Medium Development	Combination of constructed materials and vegetation. Impervious surfaces account for 50% to 79% of the total cover. These areas most commonly include single-family housing units.	Developed
High Development	Highly developed areas where people reside or work in high numbers. Examples include apartment complexes, row houses and commercial/industrial. Impervious surfaces account for 80% to 100% of the total cover.	Developed
Agriculture	Pasture/hay fields, crop lands, and perennial woody crops such as orchards and vineyards. Crop vegetation accounts for > 20% of total vegetation. This class also includes all land being actively tilled.	Altered
Open Space	Mostly vegetation in the form of lawn grasses. Commonly include large-lot single-family housing units, parks, golf courses, and vegetation planted in developed settings for recreation, erosion control, or aesthetic purposes.	Altered
Barren	Exposed areas of bedrock and other accumulations of earthen material. Generally, vegetation accounts for < 15% of total cover.	Natural
Forest	Dominated by deciduous, conifer and mixed forests	Natural
Schrub/scrub	Dominated by shrubs	Natural
Grassland	Dominated by herbaceous plants	Natural
Wetland	Dominated by water and water-dependent vegetation	Natural

Simple linear regression was used to determine the relationship between home range or core area size and percentage of unnatural land covers within each range. In particular, we conducted four regression analyses in RStudio, using the percentage of both altered and developed lands in the home range and core areas as the explanatory variable, and log-transformed space use estimates as the dependent variable.

### Resource selection

We examined 3rd-order selection [46] of land covers by coyotes utilizing resource selection functions (RSFs) to investigate use-versus-availability of the three broad land cover types, and distances to roads of each available coyote GPS fix [22]. We ran two sets of generalized linear models (GLM) using composite and seasonal home ranges in RStudio. We used coyote fixes within 95% *a*-LoCoH composite and seasonal home ranges for each model set to represent coyote use of the broad land cover types, and generated 5,000 random points in each home ranges to serve as a measure of availability [47]. Composite GLMs included sex, status, land cover, time periods, and distance to roads as explanatory variables. Seasonal GLMs included sex, status, land cover, season, and distance to roads as explanatory variables. The distance to roads of each location was log-transformed to meet distributional assumptions. For categorical variables, reference categories were resident (status), female (sex), natural (land cover), and dawn (time period). Seasonal models included season as an explanatory model with the breeding season as a reference.

### Diel activity and movement distances

We define movement distance as the minimum distance between two sequential points, separated by a duration of 1.5 h. Movement distances were calculated using the *pointdistance* function in the geospatial modelling environment (GME) version 0.7.4.0 [48]. We tested for differences in movement distance as a function of status and time periods. Descriptive statistics, *t*-tests, and ANOVA were performed in RStudio. Activity patterns of coyotes, evaluating the interaction of coyote status and time period, were illustrated using the ggplot2 version 3.1.0 package in RStudio [49].

Generalized linear mixed models (GLMM) were used to test the effect of explanatory variables on the response variable (1.5 h movement distances). Explanatory variables included status, sex, season, path (initial land cover to final land cover), and temperature as fixed effects. Average ambient temperature was obtained by individual coyote collars at each collected fix. We treated individual coyotes as a random factor to account for pseudoreplication.

For all GLM and GLMM analyses, we plotted residual vs. fitted values to verify homogeneity of variance [50], and tested for collinearity [51] using the ‘vif’ function in the ‘car’ version 2.1–6 package [52]. We used ‘lme4’ version 1.1–16 package [53] and ‘dredge’ function using ‘MuMIn’ version 1.40.4 package [54] to test all possible model combinations. For resource selection and movement models, we selected top candidate models (ΔAICc < 2) with models ranked based on Akaike Information Criterion (AIC) with a second-order correction for small sample size (AICc), which ranks models dependent on fit [55]. When GLMs or GLMMs provided more than one top model, models were averaged using ‘MuMIn’ package in order to identify key explanatory variables and their associated model coefficients.

## Results

We captured a total of 35 coyotes from October 2010 through February 2012, 28 of which (16 males 12 females) were fitted with GPS collars for the purposes of this study. Ultimately, data from 27 coyotes was used for this study after one female coyote was killed by a vehicle shortly after being collared, and data from that female was not used for analyses. However, the collar was recovered and fitted on a male coyote. The number of GPS fixes from 27 coyotes was 2666 ± 1112 (avg. ± *SD*; range: 256−4,289 locations).

### Space use estimates

We estimated 27 composite coyote *a-*LoCoH home ranges, *a-*LoCoH core areas, and 95% MCP estimates. Seasonal data provided estimates of 82 home ranges and 81 core areas for both resident and transient coyote analyses, as one core area could not be effectively calculated in RStudio. Eighty-two seasonal 95% MCP were used for space use analyses.

### Home range

Mean (± *SD*) home-range size of resident coyotes (4.7 ± 1.8 km^2^) was significantly smaller than transient coyotes (67.7 ± 89.6 km^2^; *t*_25_ = −6.20, *P* < 0.005; Table 2). Resident home range size for males (4.9 ± 2.2 km^2^) was not significantly different than females (4.5 ± 1.5 km^2^; *t*_11_ = −0.19, *P* = 0.85), nor was resident home range size of adult coyotes (4.4 ± 1.3 km^2^) significantly different than young (5.1 ± 2.5 km^2^; *t*_11_ = −0.30, *P* = 0.77). Transient home range size for males (75.3 ±102.7 km^2^) was not significantly different than females (48.5 ± 49.7 km^2^; *t*_12_ = −0.37, *P* = 0.72), nor were home ranges of transient adults (85.8 ± 113.8 km^2^) significantly different than young (49.5 ± 60.6 km^2^; *t*_12_ = 0.50, *P* = 0.63). However, adult males had noticeably large home ranges and core areas compared to other transient females and young males. Seasonal home ranges of resident coyotes averaged (± *SD*) 4.4 ± 2.2 km^2^ and did not differ significantly by season (F_2,29_ = 0.11, P = 0.90), sex (F_1,29_ = 2.06, P = 0.16), nor age (F_1,29_ = 0.36, P = 0.56) classes (Table 3).

**Table 2:** Composite home range (95% *a*-LoCoH), core area (50% *a*-LoCoH), and 95% MCP for coyotes (*Canis latrans*) radio-collared in the Cuyahoga Valley, Ohio, 2010–2012.

			95% *a*-LoCoH		50% *a*-LoCoH		95% MCP	
Status	Age-Sex	*n*	$\bar{x}$	*SD*	$\bar{x}$	*SD*	$\bar{x}$	*SD*
Resident	Adult Male	3	4.8	1.7	1.0	0.7	6.2	1.7
	Adult Female	5	4.2	1.2	0.8	0.4	5.9	1.2
	Young Male	3	5.1	3.0	1.1	1.0	6.2	3.7
	Young Female	2	5.2	2.6	0.8	0.0	7.0	4.0
Transient	Adult Male	4	115.4	144.0	21.1	25.5	306.1	458.1
	Adult Female	3	46.3	60.6	9.4	12.6	80.2	80.1
	Young Male	6	48.6	66.4	7.0	13.2	236.6	182.9
	Young Female	1	55.2	-	12.5	-	76.8	-

**Table 3:** Seasonal home range (95% *a*-LoCoH), core area (50% *a*-LoCoH), and 95% MCP for resident coyotes (*Canis latrans*) radio-collared in the Cuyahoga Valley, Ohio, 2010–2012.

Season	Age	Sex	95% *a*-LoCoH			50% *a*-LoCoH			95% MCP		
			*n*	$\bar{x}$	*SD*	*n*	$\bar{x}$	*SD*	*n*	$\bar{x}$	*SD*
Breeding	Adult	Male	3	4.6	2.7	3	1.0	0.2	3	6.6	2.7
		Female	5	4.0	1.4	5	1.5	0.7	5	33.6	50.4
	Young	Male	3	5.6	3.3	3	1.9	2.2	3	31.0	41.6
		Female	1	2.7	−	1	0.4	−	1	6.2	−
Pup-rearing	Adult	Male	3	5.0	2.6	3	1.2	0.3	3	5.0	2.2
		Female	4	4.0	1.4	4	0.5	0.3	4	3.8	0.7
	Young	Male	2	5.5	4.9	2	1.3	1.3	2	5.0	4.7
		Female	1	4.3	−	1	1.1	−	1	3.7	−
Dispersal	Adult	Male	6	4.5	1.9	5	1.4	0.3	5	5.6	2.0
		Female	7	3.1	0.5	7	1.1	0.5	7	9.5	8.6
	Young	Male	3	6.7	4.7	3	2.7	2.1	3	5.5	1.1
		Female	3	4.2	1.9	3	1.0	0.8	3	5.5	3.1

### Core area

Mean (± *SD*) core area size of resident coyotes (0.9 ± 0.6 km^2^) was significantly smaller than transient coyotes (11.9 ± 16.7 km^2^; *t*_25_ = −4.37, *P* < 0.005; Table 2). Resident core area size for males (1.1 ± 0.8 km^2^) was not significantly different than females (0.8 ± 0.3 km^2^; *t*_11_ = −0.29, *P* = 0.78), nor was resident core area size of adult coyotes (0.9 ± 0.5 km^2^) significantly different than young (1.0 ± 0.7 km^2^; *t*_11_ = 0.02, *P* = 0.98). Transient core area size for males (12.7 ± 19.1 km^2^) was not significantly different than females (10.1 ± 10.4 km^2^; *t*_12_ = 0.32, *P* = 0.76), nor was core area size of transient adults (16.1 ± 20.4 km^2^) significantly different than young (7.8 ± 12.2 km^2^; *t*_12_ = 1.15, *P* = 0.27). Seasonal core area of resident coyotes averaged (± *SD*) 1.3 ± 1.0 km^2^ and did not differ significantly by season (F_2,28_ = 1.5, P = 0.25), sex (F_1,28_ = 2.60, P = 0.19), and age (F_1,28_ = 0.04, P = 0.84) classes (Table 3).

### Minimum convex polygon estimates

Mean 95% MCP area (± *SD*) of resident coyotes (6.2 ± 2.2 km^2^) was significantly smaller than transient coyotes (211.5 ± 265.9 km^2^; *t*_25_ = −9.26, *P* < 0.005; Table 2). Resident home range size for males (6.2 ± 2.6 km^2^) was not significantly different than females (6.2 ± 2.0 km^2^; *t*_11_ = −0.20, *P* = 0.85), nor was resident home range size of adult coyotes (6.0 ± 1.3 km^2^) significantly different than young (6.5 ± 3.3 km^2^; *t*_11_ = 0.08, *P* = 0.93). Transient home range size for males (264.4 ± 299.7 km^2^) was not significantly different than females (79.4 ± 64.4 km^2^; *t*_12_ = −1.4, *P* = 0.19), nor were home ranges of transient adults (209.3 ± 348.8 km^2^) significantly different than young (213.8 ± 177.6 km^2^; *t*_12_ = −1.0, *P* = 0.34). Seasonal home ranges of resident coyotes averaged (± *SD*) 7.1 ± 11.7 km^2^ and did not differ significantly by season (F_2,29_ = 1.67, P = 0.21), sex (F_1,29_ = 3.18, P = 0.09), nor age (F_1,29_ = 2.38, P = 0.13) classes (Table 3).

### Land cover composition and use

#### Land cover composition

**Fig 2:**
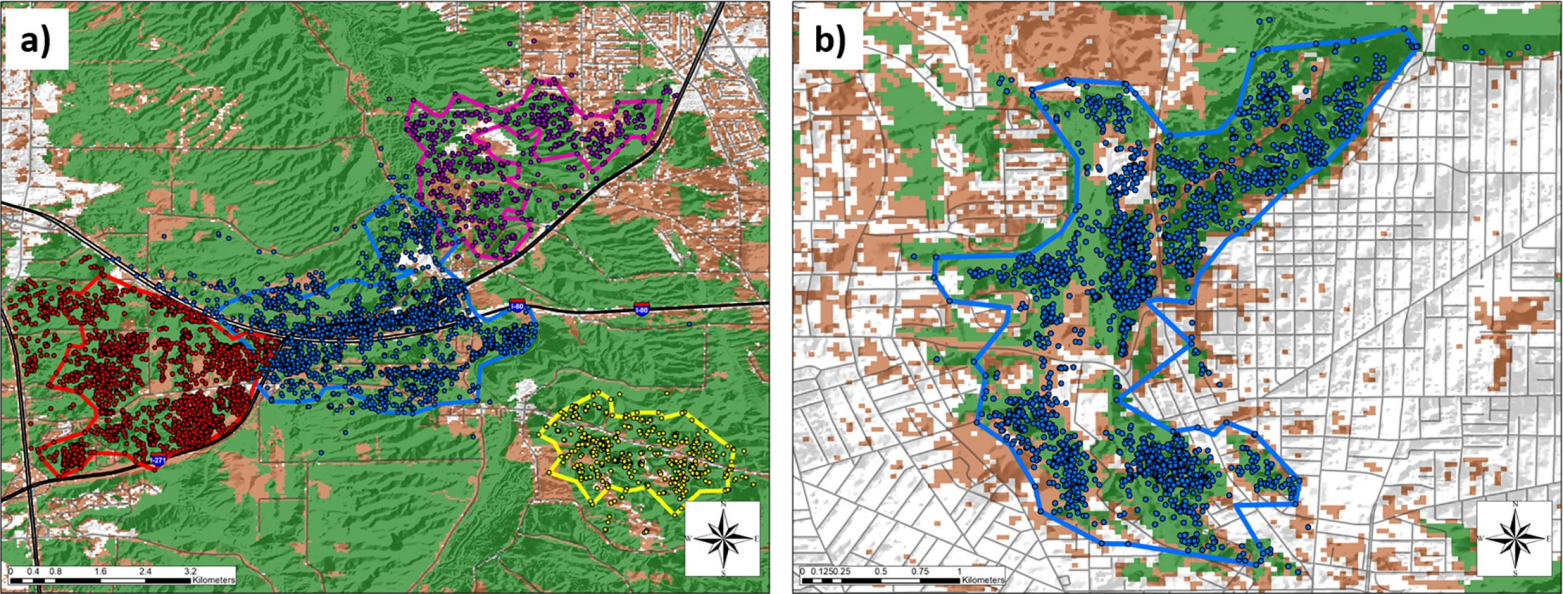
Home ranges of resident coyotes (*Canis latrans*) that are (a) fragmented and bordered by roads or (b) almost completely surrounded by developed land in the Cuyahoga Valley, Ohio, 2010–2012.

**Fig 3:**
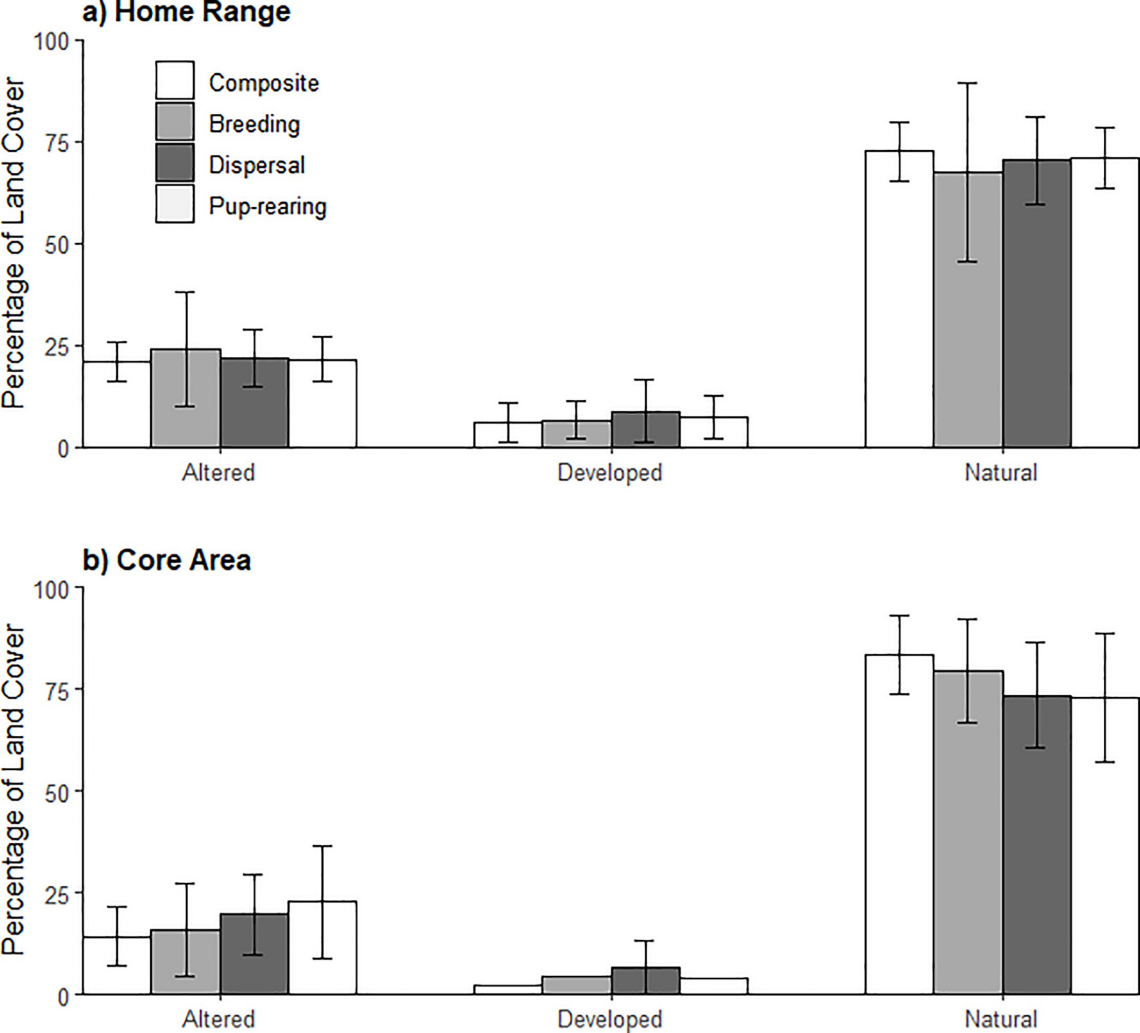
Percentage of the three broad land cover types comprising composite and seasonal resident coyote (*n* = 13) (a) home ranges and (b) core areas in the Cuyahoga Valley, Ohio, 2010–2012.

**Fig 4:**
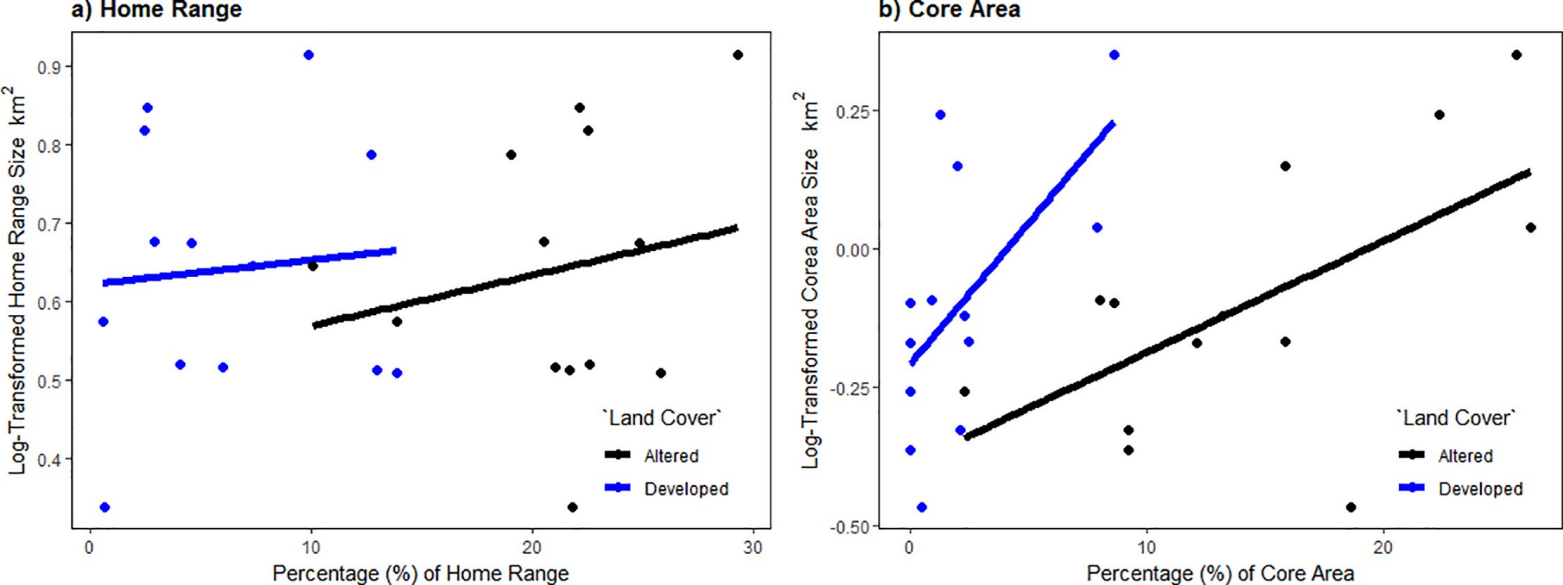
Relationship between resident coyote (*n* = 13, *Canis latrans*) composite (a) home range and (b) core area size and the percentage of altered and developed land cover types within, Cuyahoga Valley, Ohio, 2010–2012.

Home range and core areas of resident coyotes were typically associated with natural land cover, with some ranges defined or fragmented by roads (Fig 2A), or nearly surrounded by developed land (Fig 2B). Natural land cover comprised an average of 72.6 ± 7.3% (± *SD*) and 71.2 ± 9.1% of composite and seasonal home ranges, respectively, with no significant differences between seasons (F_2,38_ = 0.30, P = 0.74; Fig 3). Altered lands comprised an average of 21.2 ± 4.9% and 21.4 ± 6.0% (± *SD*) of composite and seasonal home ranges, respectively, with no significant differences between seasons (F_2,38_ = 0.25, P = 0.78). Developed lands comprised an average of 6.2 ± 4.7% and 7.4 ± 5.7% (± *SD*) of composite and seasonal home ranges, respectively, with no significant differences between seasons (F_2,38_ = 0.12, P = 0.89). The percentage of altered land cover was not related to composite (F_1,11_ = 0.59, P = 0.46, R^2^ = −0.036, Fig 4A) or seasonal home range size (F_1,39_ = 0.24, P = 0.62, R^2^ = −0.019). Developed land cover was also not related to composite (F_1,11_ = 0.09, P = 0.77, R^2^ = −0.082, Fig 4A) or seasonal home range size (F_1,39_ = 0.07, P = 0.79, R^2^ = −0.023).

We found natural lands comprised an average of 83.4 ± 9.6% and 75.4 ± 13.7% of (± *SD*) composite and seasonal core area, respectively, with no significant differences between seasons (F_2,37_ = 0.83, P = 0.44; Fig 3). Altered lands comprised an average 14.4 ± 7.2% and 19.4 ± 11.4% (± *SD*) of composite and seasonal core areas, respectively, with no significant differences between seasons (F_2,37_ = 1.00, P = 0.38). Developed lands comprised an average of 2.2 ± 2.9% and 5.5 ± 6.0% (± *SD*) of composite and seasonal core areas, respectively, with no significant differences between seasons (F_2,37_ = 0.75, P = 0.48). The percentage of altered land cover was positively correlated to composite core areas (F_1,11_ = 6.48, P = 0.03, R^2^ = 0.313, Fig 4B), but there was no relation for seasonal core areas (F_1,38_ = 3.13, P = 0.08, R^2^ = 0.52). Developed land cover was positively correlated to composite (F_1,11_ = 6.44, P = 0.03, R^2^ = 0.313, Fig 4B) and seasonal core areas (F_1,38_ = 9.70, P < 0.005, R^2^ = 0.182).

#### Land cover use

For all coyotes, use of natural land cover (84.9 ± 11.8%; ± *SD*) was significantly greater (F_2,825_ = 6440.0, P < 0.005) than altered (13.6 ± 10.8; ± *SD*) and developed land (1.4 ± 2.1%; ± *SD*; Table 4). The use of natural lands was used significantly higher for all time periods (F_6,816_ = 20.53, P < 0.005) with no significant differences in use of land cover types between seasons (F_4,810_ = 1.63, P = 0.17) or status (F_2,810_ = 1.83, P = 0.16). Higher percentages of nocturnal coyote locations were found within altered and developed land covers when compared to diurnal locations.

**Table 4:** Percentage of coyote (n = 27 individuals, *Canis latrans*) locations found within the three broad land cover categories throughout diel periods in the Cuyahoga Valley, Ohio, 2010–2012.

		Time Period				
Season	Land cover type	Dawn	Day	Dusk	Night	Overall
Breeding	Natural	85.8	91.3	83.3	78.0	83.2
	Altered	12.7	8.0	14.9	18.9	14.8
	Developed	1.5	0.7	1.8	3.1	2.0
Dispersal	Natural	89.0	91.7	85.5	78.4	83.6
	Altered	10.3	8.0	13.4	19.2	14.8
	Developed	0.7	0.3	1.1	2.4	1.6
Pup-rearing	Natural	89.2	90.1	80.5	77.5	85.5
	Altered	9.8	9.3	17.2	19.1	13.0
	Developed	1.0	0.7	2.3	3.4	1.6

For all coyotes, the average (± *SD*) distance of coyote locations to the nearest road was 262.2 ± 196.8 m, with a mean distance to roads of resident coyotes (226.8 ± 150.9 m) significantly less than transients (303.2 ± 232.6; *t*_719863 =_ − 39.6, P < 0.005). For resident coyotes, mean distance to the nearest road within time periods were 234.5 ± 152.4 m during dawn, 228.5 ± 146.4 m during the day, 230.3 ± 150.0 m during dusk, and 221.9 ± 152.8 m at night, where residents were significantly closer to roads during the night than during other time periods (F_3,38621_ = 60.2, P < 0.005). For transient coyotes, mean distance to the nearest road within time periods was 306.6 ± 232.4 m during dawn, 319.3 ± 246.9 m during the day, 310.0 ± 228.9 m during dusk, and 293.2 ± 226.7 m at night. Transients were also significantly closer to roads during the night than the other time periods (F_3,33359_ = 29.4, P < 0.005).

### Resource selection

**Fig 5:**
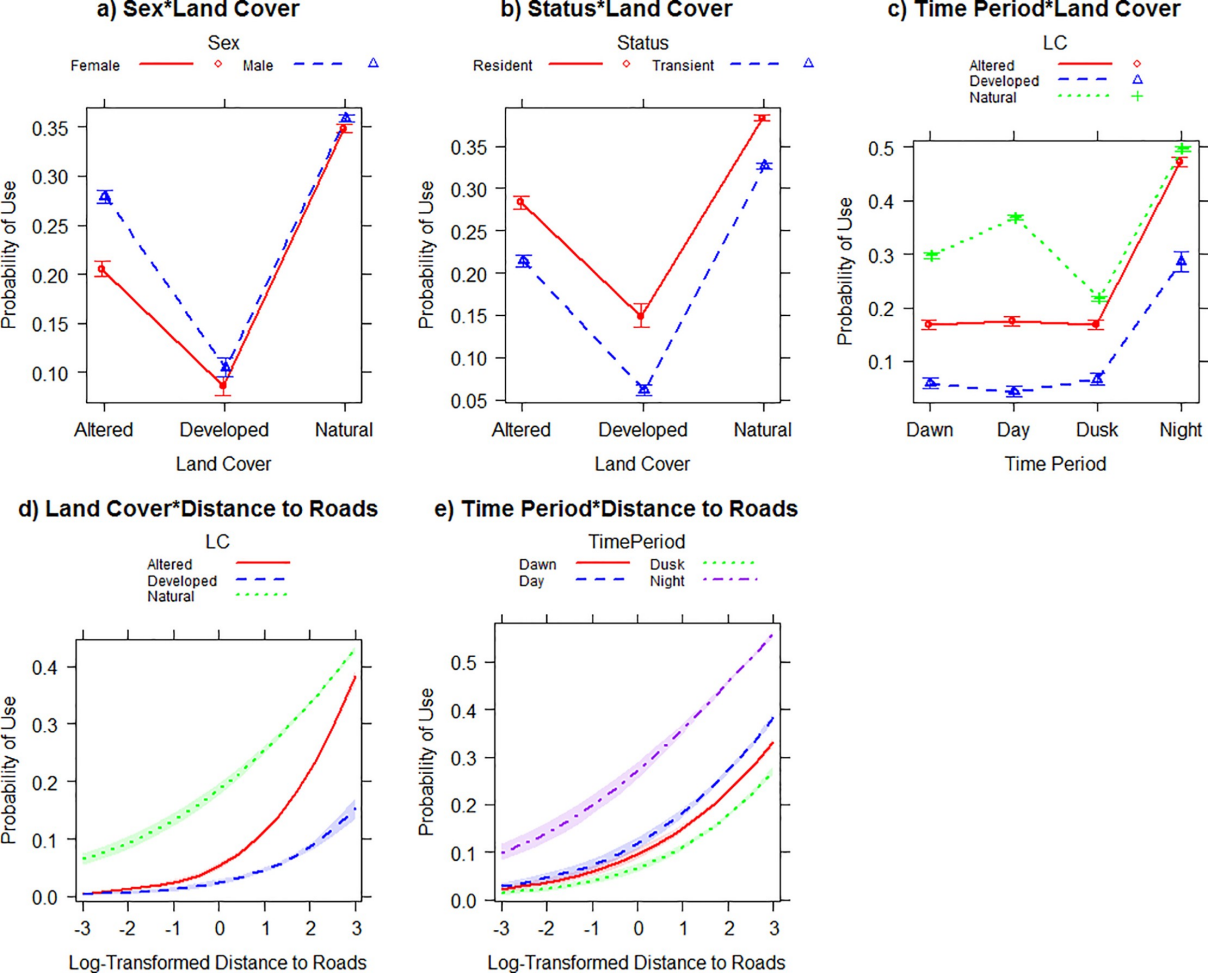
Interaction plots of a) Sex*Land Cover, b) Status*Land Cover, c) Time Period*Land Cover, d) Land Cover* Distance to Roads, and e) Time Period*Distance to Roads for the top model in the composite home range model set, of coyotes (*Canis latrans*) radio-collared in the Cuyahoga Valley, Ohio, 2010–2012.

The best-fitting candidate composite model included all explanatory variables and their interactions, excluding Status*Roads; however, the competing second model (AIC_c_ < 2) included all interactions (Tables 5 and 6). In the composite model, males and females exhibited no difference in the use of the land cover types, where use of developed land was less than altered and natural land (Fig 5A). Resident coyotes used developed land more than transient coyotes, but both groups used altered and natural land more than developed (Fig 5B). For all coyotes, natural lands had a higher probability of use throughout all time periods (Fig 5C). The use of all land cover types increased during nocturnal periods, with the use of altered and natural lands being used similarly during the day and night, while developed lands were used the least (Fig 5C). For all coyotes, locations close to roads were more likely to be within natural landscapes, with locations in altered land having roads approximately 100 m away (Fig 5D). For all coyotes, roads were used similarly throughout time periods. However, locations closer to roads had a higher probability of occurring at night than any other time periods (Fig 5E).

**Table 5:** Top models representing resource selection within composite and seasonal home ranges of coyotes (*Canis latrans*) radio-collared in the Cuyahoga Valley, Ohio, 2010–2012. Rows shown in bold indicate top models (ΔAICc < 2), df = degrees of freedom, ΔAICc = deviation for AICc compared with top model, weight = AICc weight.

Model		df	ΔAIC_c_	ΔAIC_c_	Weight
Composite Models:					
	Sex, Status, Time Period, Land Cover, Roads, Sex*Time Period, Status*Time Period, Sex*Land Cover, Status*Land Cover, Time Period*Land Cover, Sex*Roads, Time Period*Roads, Land Cover*Roads	**31**	**237367.5**	**0.00**	**0.451**
	Sex, Status, Time Period, Land Cover, Roads, Sex*Time Period, Status*Time Period, Sex*Land Cover, Status*Land Cover, Time Period*Land Cover, Sex*Roads, Status*Roads, Time Period*Roads, Land Cover*Roads	**32**	**237367.5**	**0.06**	**0.437**
	Sex, Status, Time Period, Land Cover, Roads, Status*Time Period, Sex*Land Cover, Status*Land Cover, Time Period*Land Cover, Sex*Roads, Status*Roads, Time Period*Roads, Land Cover*Roads	29	237371.7	4.18	0.056
Seasonal Models					
	Sex, Status, Season, Land Cover, Roads, Sex*Land Cover, Status*Land Cover, Season*Land Cover, Sex*Roads, Status*Roads, Season*Roads, Land Cover*Roads	**22**	**367622.5**	**0.00**	**1.000**
	Sex, Status, Season, Land Cover, Roads, Sex*Land Cover, Status*Land Cover, Season*Land Cover, Status*Roads, Season*Roads, Land Cover*Roads	21	367673.6	51.07	0.000
	Sex, Status, Season, Land Cover, Roads, Sex*Land Cover, Status*Land Cove, Season*Land Cover, Sex*Roads, Status*Roads, Season*Roads, Land Cover*Roads	18	367684.8	62.27	0.000

**Table 6:** Beta coefficients of the top models (ΔAIC_c_ < 2) in composite model sets used to determine land cover selection of coyotes (*Canis latrans*) radio-collared in the Cuyahoga Valley, Ohio, 2010–2012.

	β	*SE*	*z*	*P*-value
(Intercept)	-3.21	0.09	33.92	< 0.001
Sex-Male	-0.11	0.06	1.63	0.102
Status-Transient	-0.33	0.06	5.21	< 0.001
Land Cover-Developed	-0.43	0.16	2.75	0.006
Land Cover-Natural	1.79	0.08	23.56	< 0.001
Time Period-Day	0.15	0.09	1.58	0.113
Time Period-Dusk	-0.05	0.10	0.53	0.598
Time Period-Night	1.71	0.08	20.63	< 0.001
Roads	0.69	0.04	17.19	< 0.001
Sex-Male*Land Cover-Developed	-0.19	0.08	2.44	0.015
Sex-Male*Land Cover-Natural	-0.36	0.03	11.78	< 0.001
Status-Transient*Land Cover-Developed	-0.61	0.08	8.10	< 0.001
Status-Transient*Land Cover-Natural	0.12	0.03	4.09	< 0.001
Sex-Male*Time Period-Day	-0.01	0.03	0.47	0.639
Sex-Male*Time Period-Dusk	-0.05	0.03	1.43	0.152
Sex-Male*Time Period-Night	-0.08	0.03	2.80	0.005
Status-Transient*Time Period-Day	-0.18	0.03	5.75	< 0.001
Status-Transient*Time Period-Dusk	0.02	0.03	0.57	0.571
Status-Transient*Time Period-Night	0.12	0.03	4.37	< 0.001
Time Period-Day*Land Cover-Developed	-0.38	0.15	2.57	0.010
Time Period-Day*Land Cover-Natural	0.27	0.05	5.73	< 0.001
Time Period-Dusk*Land Cover-Developed	0.13	0.13	0.99	0.322
Time Period-Dusk*Land Cover-Natural	-0.43	0.05	8.95	< 0.001
Time Period-Night*Land Cover-Developed	0.37	0.11	3.41	0.001
Time Period-Night*Land Cover-Natural	-0.65	0.04	16.35	< 0.001
Sex-Male*Roads	0.25	0.03	9.87	< 0.001
Status-Transient*Roads	-0.02	0.03	0.69	0.488
Land Cover-Developed*Roads	-0.15	0.06	2.52	0.012
Land Cover-Natural*Roads	-0.41	0.03	13.66	< 0.001
Time Period-Day*Roads	0.00	0.04	0.02	0.981
Time Period Dusk*Roads	0.04	0.04	0.88	0.379
Time Period-Night*Roads	-0.11	0.03	3.15	0.002

**Fig 6:**
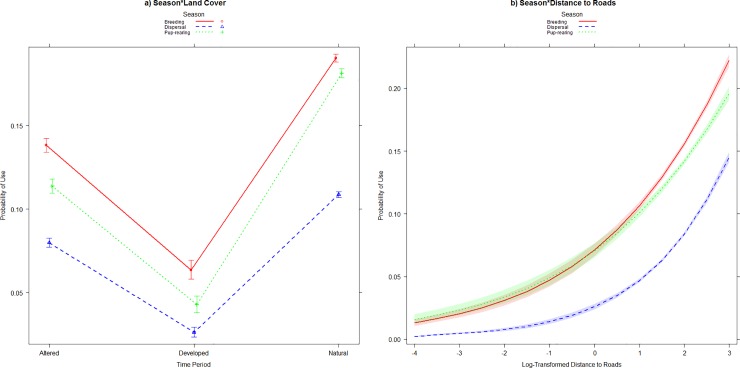
Interaction plots of a) Season*Land Cover, and b) Season*Distance to Roads for the top model in the seasonal home range model set of coyotes (*Canis latrans*) radio-collared in the Cuyahoga Valley, Ohio, 2010–2012.

In the seasonal model set, the global model with all model weights was the top model with 100% of the model weights (Tables 5 and 7). Seasonally, we found no differences in land cover selection. For all coyotes, land cover types were not used differently between the three seasons (Fig 6A). Seasonal results of land cover use among sex and status exhibited similar patterns to composite models with natural and altered lands used more than developed land. Coyotes used land cover types in relation to roads similarly among seasons, with a higher probability of locations falling closer to roads in natural and altered lands than in developed land (Fig 6B).

**Table 7:** Beta coefficients of the top models (ΔAIC_c_ < 2) in the seasonal model sets used to determine land cover selection of coyotes (*Canis latrans*) radio-collared in the Cuyahoga Valley, Ohio, 2010–2012.

	β	*SE*	*z*	*P*-value
(Intercept)	-3.62	0.07	-52.62	< 0.001
Sex-Male	-0.02	0.05	-0.44	0.659
Status-Transient	0.48	0.05	9.21	< 0.001
Land Cover-Developed	0.07	0.12	0.62	0.538
Land Cover-Natural	1.28	0.06	21.05	< 0.001
Season-Dispersal	-1.00	0.06	-16.50	< 0.001
Season-Pup-rearing	-0.11	0.06	-1.86	0.063
Roads	0.79	0.03	26.22	< 0.001
Sex-Male*Land Cover-Developed	-0.02	0.07	-0.25	0.800
Sex-Male*Land Cover-Natural	-0.33	0.03	-12.75	< 0.001
Status-Transient*Land Cover-Developed	-0.47	0.07	-6.98	< 0.001
Status-Transient*Land Cover-Natural	0.13	0.02	5.17	< 0.001
Season-Dispersal*Land Cover-Developed	-0.31	0.08	-4.01	< 0.001
Season-Dispersal*Land Cover-Natural	-0.04	0.03	-1.54	0.124
Season-Pup-rearing*Land Cover-Developed	-0.19	0.08	-2.41	0.016
Season-Pup-rearing*Land Cover-Natural	0.16	0.03	5.35	< 0.001
Sex-Male*Roads	0.16	0.02	7.29	< 0.001
Status-Transient*Roads	-0.34	0.02	-15.64	< 0.001
Land Cover-Developed*Roads	-0.32	0.05	-6.07	< 0.001
Land Cover-Natural*Roads	-0.35	0.03	-13.92	< 0.001
Season-Dispersal*Roads	0.18	0.03	7.03	< 0.001
Season-Pup-rearing*Roads	-0.05	0.03	-1.93	0.053

### Diel activity and movement

GPS collars fitted on resident and transient coyotes provided 36,177 suitable 1.5 h movement distances. The mean (± *SD*) 1.5 h movement distance for all coyotes was 415.8 ± 586.3 m (range 0.0–6199.2 m), with resident coyote movement distances (460.3 ± 576.7 m) significantly greater than transient (363.6 ± 593.2 m; *t*_36175_ = 21.8, P < 0.005). Resident coyote male movement distances averaged 475.8 ± 596.3 m (± *SD*) and were significantly greater than female movement distances (442.7 ±552.9 m); *t*_19537_ = −2.7, *P* = 0.007). Similarly, transient male coyote movement distances (380.5 ± 613.8 m) were significantly greater than female transients (332.7 ± 552.3 m; *t*_16636_ = −6.9, *P* < 0.001).

**Fig 7:**
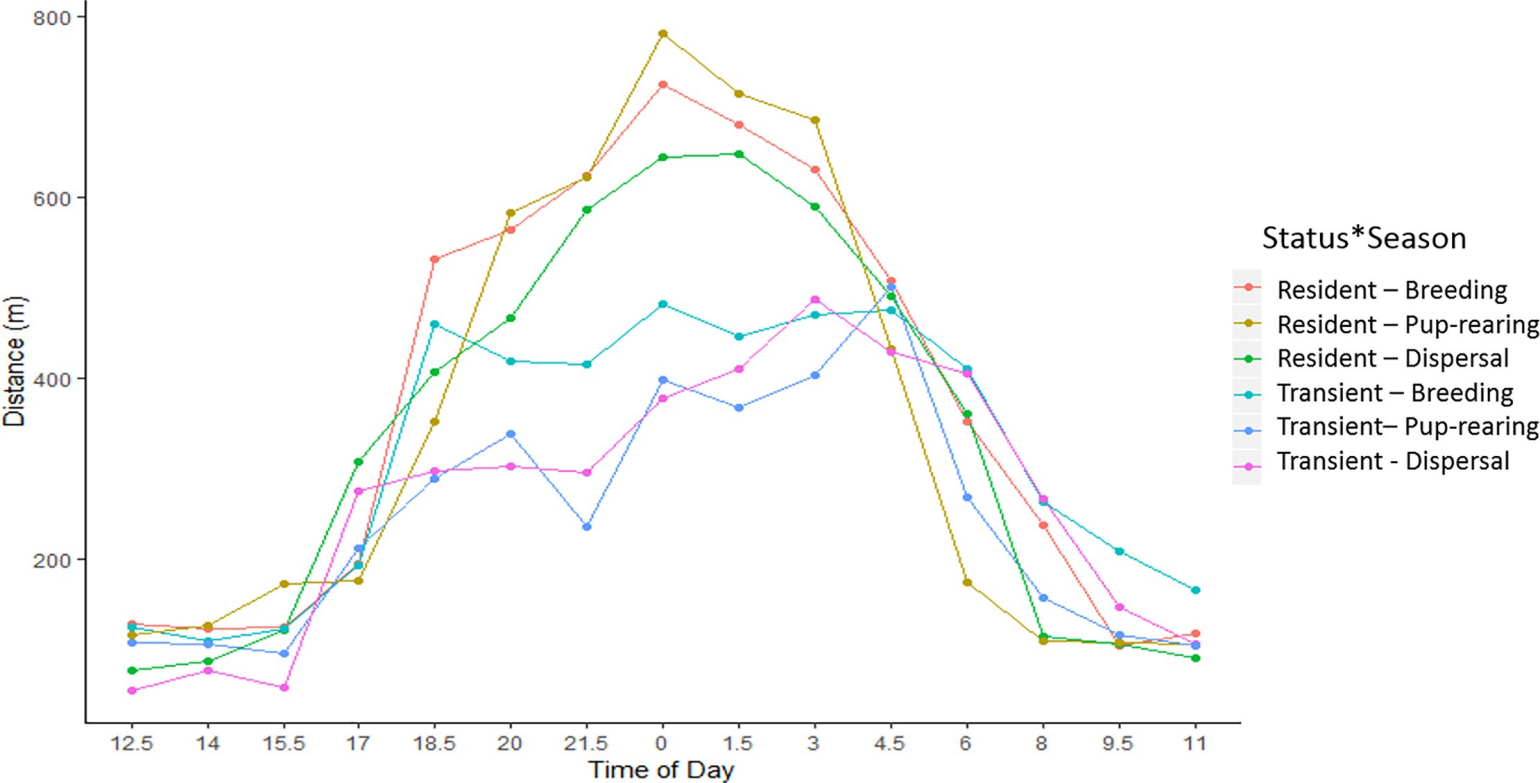
Seasonal activity patterns of GPS collared resident and transient coyotes (*Canis latrans*) in the Cuyahoga Valley, OH, 2010–2012.

Resident coyote movement distances averaged (± *SD*) 206.3 ± 343.3 m at dawn, 156.1 ± 294.9 m during the day, 575.2 ± 619.6 m at dusk, and 574.8 ± 615.9 m during the night. Transient coyote distances average 324.6 ± 515.0 m at dawn, 147.6 ± 312.9 m during the day, 319.6 ± 501.6 m at dusk, and 428.9 ± 653.1 at night. Resident and transient coyotes were more active during the night. However, transient coyote movement was significantly less than residents during the night for all biological seasons (F_6,36153_ = 2.6, P = 0.02; Fig 7).

The best-fitting candidate movement model (AIC_c_ < 2) included all explanatory variables (*i*.*e*. sex, status, season, time period, path, and temperature) and their interactions, having 64% of the model weight (Table 8 and Table 9). The movement distances of females and male coyotes were similar, but movement distance by transient coyotes was less in a 1.5 h period than resident coyotes. Movement distances of coyotes were less when travelling within the same land cover type, with movements beginning and ending in developed areas being less than all others (S1 Fig). For all coyotes, movement distances exhibited a negative relationship with ambient temperature, showing less movement as a result of increased temperatures.

**Table 8:** Top models showing results from top GLMMs comparing model fitness for 1.5 h movement distances of coyotes (*Canis latrans*) radio-collared in the Cuyahoga Valley, Ohio, 2010–2012. Rows shown in bold indicate top models (ΔAICc < 2), df = degrees of freedom, ΔAICc = deviation for AICc compared with top model, weight = AICc weight.

Model	df	ΔAIC_c_	ΔAIC_c_	Weight
**Status + Season + Time Period + Path + Temperature**	**18**	**78045.0**	**0.0**	**0.641**
**Sex + Status + Season + Time Period + Path + Temperature**	**19**	**78046.9**	**1.95**	**0.241**
Time Period + Path + Temperature	17	78049.1	4.09	0.083
Sex + Season + Time Period + Path + Temperature	18	78050.8	5.82	0.035

**Table 9:** Beta coefficients of the top models (ΔAIC_c_ < 2) in the movement model set used to predict movement distances of coyotes (*Canis latrans*) radio-collared in the Cuyahoga Valley, Ohio, 2010–2012.

	β	*SE*	*z*	*P*-value
(Intercept)	2.24	0.05	48.24	< 0.001
Sex-Male	0.01	0.06	0.22	0.82
Status-Transient	-0.14	0.05	2.61	0.01
Sex-Male	0.01	0.06	0.22	0.82
Status-Transient	-0.14	0.05	2.61	0.01
Season-Dispersal	0.15	0.01	14.63	< 0.001
Season-Pup-rearing	0.61	0.01	47.01	< 0.001
Time Period-Day	0.09	0.02	5.53	< 0.001
Time Period-Dusk	0.35	0.02	19.52	< 0.001
Time Period-Night	0.20	0.02	13.29	< 0.001
Path-Altered to Developed	0.76	0.06	13.12	< 0.001
Path-Altered to Natural	0.78	0.02	38.99	< 0.001
Path-Developed to Altered	0.74	0.05	14.02	< 0.001
Path-Developed to Developed	-0.14	0.06	2.15	0.03
Path-Developed to Natural	0.79	0.04	20.54	< 0.001
Path-Natural to Altered	0.82	0.02	39.86	< 0.001
Path-Natural to Developed	0.88	0.04	21.66	< 0.001
Path-Natural to Natural	0.18	0.02	11.51	< 0.001
Temperature	-0.06	0.00	78.81	< 0.001

## Discussion

Variation in coyote home range size is apparent across North America [34,56–59] and may be influenced by habitat composition [7,20,36]. Radiotelemetry studies have quantified coyote space use in relatively rural and natural areas [34,36,60,61], ranching and agricultural areas [57,62–64], and urbanized areas [6,7,19,20]. Home range size of resident coyotes (4.7 ± 1.8 km^2^, range: 2.2–8.2 km^2^) in our study did not differ from urban resident coyote home ranges in Chicago and Los Angeles (approximately 5 km^2^), but were less than half the size of urban coyotes (11–13 km^2^) in Tucson, AZ and Lower Fraser Valley, BC (see Table 7.2 in [12]). Similar to other urban studies, resident coyote home ranges were smaller than that of transient coyotes [5,7,22].

We found no differences in home range or core areas as a function of sex for resident or transient coyotes, which was similar results to other studies that compared home range sizes between sex in both urbanized and rural environments [7,22,36,57,61]. Contrary to our study, Riley et al. [20] found that males have significantly larger home ranges than females in southern California, whereas Holzman et al. [58] and Chamberlain [65] detected larger home ranges in females than males. Resident coyote space use in the Cuyahoga Valley did not differ by season. Other urban studies have found no seasonal variation in space use [7,22]; however, Gese et al. [5] found seasonal variation in home ranges based on levels of human development in the Chicago metropolitan area, and Grubbs and Krausman [6] found home range sizes of resident coyotes to be significantly smaller during the dispersal period.

Small home ranges and core areas of resident coyotes may indicate high population densities [7,66], resulting from reduced mortality from hunting or trapping, and/or increased food availability, or both [12]. Reports of coyote diets in urban landscapes have shown consumption of both natural and anthropogenic foods [9–14]. Diet studies conducted in northeast Ohio (Cleveland Metroparks and Cuyahoga Valley National Park) suggest that small mammals (e.g. *Miscrotus sp*.), white-tailed deer (*Odocoileus virginianus*), eastern cotton tail rabbit *(Sylvilagus floridanus*) and raccoons (*Procyon lotor*) comprised the largest portions of natural food, with little evidence of anthropogenic food [67,68]. However, coyotes may still utilize areas that could potentially attract prey species in human-dominated locations composed of manicured lawns, trash collections, and buildings that serve as cover.

Similar to Gehrt et al. [7] in Chicago and Riley et al. [20] in southern California, home range of resident coyotes in the Cuyahoga Valley were comprised of high percentages of natural land covers and small percentages of developed lands (home range: 0–14%; core area: 0–9%). This may suggest avoidance of these land cover types. In contrast, where developed lands spanned 62% of the Denver metropolitan area, Poessel et al. [22] reported urban coyote home ranges were comprised of 44% developed land. Within our study area, altered and developed lands only accounted for 24.2% and 18.9% of available land cover, respectively, and therefore percentages within coyote spaces remained small. We found a significant, positive relationship between core area size and availability of altered and developed land cover types, suggesting coyotes may need more space at that scale. In the Chicago metropolitan area, coyotes in developed areas had home ranges twice the size of individuals in less-developed areas [5]. Coyotes in our study did spend more time in natural landscapes, which is common in urbanized landscapes [5,7,8,16,21,22]. In particular, coyotes spent more time in natural land covers throughout all periods of the day, with higher use of altered and developed lands during crepuscular and nighttime hours. Much of the natural lands in the Cuyahoga Valley are comprised of dense deciduous forest that offers opportunity for den placement, provides shade during the heat of the summer, cover from precipitation in the winter, and refuge from humans. Assuming then that coyotes are acquiring most of their resource needs from natural landscapes, as those landscapes become more fragmented by anthropocentric habitats, coyotes have to venture further to fulfill their resource needs.

Transients comprised 52% of coyotes in this study. Subadult canids typically disperse from their natal territory covering large tracts of land, possibly as a result of poor-quality habitat, widely dispersed resources, or searches for available territories [5,36]. It can be expected that higher numbers of resident coyotes inhabit the natural lands of the Cuyahoga Valley and our similar resident-transient numbers is a possibly a result of available trapping locations. Surrounded by urbanization, natural areas along the Cuyahoga River may serve as an important corridor for transient coyotes to move themselves throughout the landscape. However, a genetic study on coyote haplotypes within Cleveland, OH and the Cuyahoga Valley reported that coyote populations in this area do not freely intermix and are partially isolated [69]. Though less-likely, it could be speculated we observed high numbers of transient coyotes stemming from the Cuyahoga Valley as a result of limited space and resources, with all available land occupied by resident coyote territories. Although we cannot discern if transient coyotes were born in the initial study area or travelling through at the time of capture, it could be suggested that this area may serve as a source for expanding populations.

Coyote resource selection in our system included all explanatory variables in our composite and seasonal model sets. Our RSF models suggest that coyotes in the Cuyahoga Valley selected natural and altered land cover types more than developed lands. This has been reported in previous urban coyote studies [5,19,21,22]. Overall, developed lands were typically selected least frequently of the three land cover types, suggesting that these areas may have the least value to coyotes. Altered land cover was selected more than developed land and less than natural lands. Way et al. [23] found that coyotes in suburban Cape Cod, MA moved through altered lands more than residential areas and natural area. Selection of altered lands may provide foraging opportunities for coyotes but run a higher risk of human detection than natural lands. Consistent with temporal use of the three land cover types, coyotes selected natural land covers more throughout the day. A decreased sense of security around humans may explain the use of developed areas mostly during the night, which we observed in our system [20]. In the Chicago metropolitan area, Gehrt et al. [7] reported that urban areas were avoided during both diurnal and nocturnal periods. However, in Tucson, Arizona high percentages of coyote locations were observed in developed lands during crepuscular and nocturnal periods.

Movement distances of coyotes in our system varied as a function of sex and status. These results differ from other urban coyotes that did not differ by sex [16,23,70] or status [70]. Movement distances of transient coyotes in our system were significantly less than residents during the crepuscular and nocturnal times when overall coyote activity was the greatest. Transient coyotes are reported to scavenge more than residents, and use less attractive habitat in order to avoid contact with residents which may explain the differences in movement distances [34,64,71–73]. It is likely that transients travel slower and cautiously through areas with higher risk of exposure to human activity in order to evade residents in our study area, as residents can exhibit aggressive behavior towards intruders [71]. Resident coyotes in our study may travel extensively throughout the nocturnal and crepuscular periods in order to defend their territories.

Our predictions were correct, and in accordance with other research [6,16,20,23,70], that coyotes in our study exhibited a nocturnal and crepuscular pattern of activity. It has been suggested that higher levels of coyote activity during crepuscular times periods could be a thermoregulatory strategy to conserve water and energy [74], which may explain why movement distance exhibited a negative relationship with ambient temperature. However, as reported with other wildlife species [75–77], we suggest this modified temporal behavior is likely avoidance of human activity and associated mortality risk (e.g. hunting, vehicular collision). Wallace [78] reported the activity patterns of coyotes near park trails in relation to human activity on recreational trails in the Cuyahoga Valley. The findings of [78] suggest resident coyotes move further away from trails during the day when human activity is highest and in closer proximity during nocturnal hours, though variation among resident individuals existed. However, transient coyotes in this system were reported to be closer to trails during the day than resident coyotes [78]. We suspect that coyotes throughout our system, not just in relation to recreational trails, exhibit this nocturnality behavior to avoid humans, especially at locations in close proximity to developed lands.

## Conclusions

In the Cuyahoga Valley of northeast Ohio, coyote activity is primarily confined to natural land cover types and limited to the nocturnal and crepuscular time periods. Such conditions correspond to the places and times where more prey species are likely available and when human activity is reduced. Because *C*. *latrans* is frequently found in urban landscapes across its range, our results contribute to the understanding of the interactions between this opportunistic species and humans along the natural/developed interface. Coyotes in the Cuyahoga Valley exhibited similar land cover preferences as those in more urbanized areas, selecting more natural land cover over unnatural. Furthermore, space use composition of coyotes were similar to other studies in urban areas, as some individual home range and core area had no developed area within them [7,20]. Our results indicate that coyotes in the Cuyahoga Valley, although surrounded by human development, tend to actively avoid such areas.

In order to maintain coyote populations and reduce opportunities for negative human coyote conflicts, land managers should consider maintaining and even expanding the abundance of natural and protected lands in the Cuyahoga Valley. Such a strategy would preserve the natural behavior of coyotes for habitat choice rather than forcing them into more urbanized landscapes where the bulk of the human population is located. Though our results indicate a high number of transient coyotes, it may not accurately reflect coyote dynamics in the Cuyahoga Valley. However, residents may be more predictable, and therefore more easily managed, than transients. Therefore, expanding the abundance of natural lands in this region may decrease the number of transients and subsequently reduce the opportunity for interactions between coyotes, humans, and pets. An increase of developed land in the area may reduce needed resources (e.g., natural denning areas, water sources, foraging areas) or deplete prey species, again forcing coyotes to find these resources in human-dominated landscapes. Coyotes in urban areas may have to compensate for reduced daytime foraging activity by increasing their foraging opportunity during crepuscular and nocturnal time periods [70]. We can reasonably expect that if development continues to infringe on natural areas, human-coyote interactions will become more likely. Although park management is often challenging, recognizing where and when coyotes are located allows managers to be forward-thinking in future plans that attempt to balance the needs of wildlife species with the recreational needs, and safety, of people.

## Supporting information

S1 Fig Movement distances between land cover types of GPS collared resident and transient coyotes (*Canis latrans*) in the Cuyahoga Valley, OH, 2010–2012. (TIFF)

S1 File Locations of GPS collared coyotes (*Canis latrans*), 2010–2012. (CSV)
