# Supplementary figures and images for: Spatial ecology of coyotes in the urbanizing landscape of the Cuyahoga Valley, Ohio
Source: PLoS One. 2019 Dec 30;14(12):e0227028. doi: 10.1371/journal.pone.0227028 (PMC6936805; doi:10.1371/journal.pone.0227028)

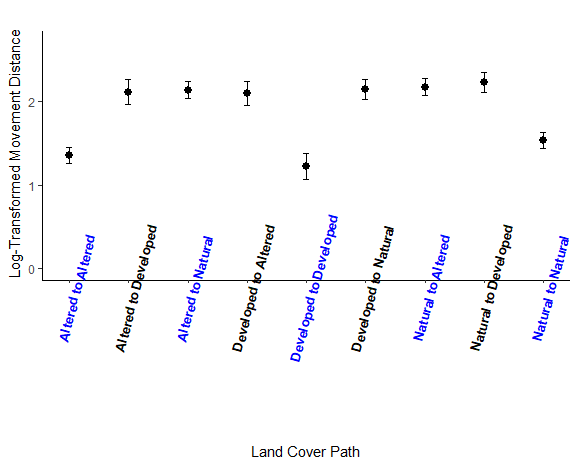

Supplement: S1 Fig — (TIFF) [file pone.0227028.s001.tiff]
